# Multimodal Analysis of Stories Told by Mental Health Influencers on TikTok

**DOI:** 10.1111/hex.70226

**Published:** 2025-04-24

**Authors:** Alex Christiansen, Shioma‐Lei Craythorne, Paul Crawford, Michael Larkin, Aalok Gohil, Spencer Strutt, Ruth Page

**Affiliations:** ^1^ University of Birmingham Birmingham UK; ^2^ Aston University Birmingham UK; ^3^ University of Nottingham Nottingham UK; ^4^ The McPin Foundation London UK

**Keywords:** influencers, mental health literacy, narrative analysis, social media, visual analysis

## Abstract

**Background:**

Social media influencers are powerful storytellers who function as conduits of public health communication and may contribute significantly to young people's mental health literacy. Influencers who discuss mental health include health professionals, wellness practitioners and experts by lived experience. As yet, there has been no multimodal analysis of how these three influencer types narrate mental health issues. This study critically evaluates 398 TikTok videos to show how three distinct types of influencers construct multimodal narratives around mental health.

**Methods:**

Data was collected using the TikTok Research API and annotated for narrative patterns and visual formatting using an inductively created multimodal framework.

**Results:**

The analysis shows important differences between the storytelling practices of health professionals, who inform others through talking head explainers, enactments and stitches, and lived experience influencers who invited shared perspectives on their stories of illness, treatment and recovery through compilations and ‘watch as I do this’ formats. Wellness practitioners occupy an interdiscursive mid‐space, blending the verbal aspects of ‘informing’ (explainers) with the visual narration of ‘shared experience’ to promote solutions through recommendation and advertising. The data also highlights similarities between the health professionals and wellness influencers in their use of marketing calls to action, indicating the commercialisation of mental health solutions offered in TikTok videos.

**Conclusions:**

It is concerning that the gap between information and support provided on TikTok may lead to partial and imbalanced development of mental health literacy by adolescent users and that content provided by certain influencer types mimics authoritative and authentic communication but promotes non‐medical solutions to mental health, unsupported by evidence.

**Patient or Public Involvement:**

Twelve young people with lived experience of mental health challenges, aged between 16 and 25, were recruited through The McPin Foundation to form the young people's advisory group (YPAG) for the project. This age range incorporates adolescents and ‘emerging adults’ who are likely to experience a range of life transitions and encounter challenges in mental health. The group met remotely four times during the study, helping to define the categories of influencers, refining the narrative categories and visual formats for the code book and discussing data examples openly to guide the analysis. Two members of the YPAG were trained and participated as coders in the inter‐rater reliability process.

## 1 Introduction

Social media has become key in sharing information at a time when over half of the young people in the UK aged 17–23 years have reported deterioration in their mental health [1] and a third of children are ‘resorting to “social media DIY” for help’ [2]. A recent systematic review shows that early and appropriate help‐seeking is obstructed by poor knowledge about mental health and where to seek help, perceived stigma and distrust of professionals [3]. Understanding how social media content is shaping mental health literacy in this very real emergency is of paramount importance. In this paper, we focus on social media influencers as informal, powerful storytellers about mental health and well‐being.

Scholars have defined influencers as ‘third party endorsers or opinion leaders’ [4] who capitalise on the potential of digital influence as a ‘commodity for the social media age’ [5]. More recently, the persuasive impact associated with influencers has begun to consider their role in shaping public health communication [6] and promoting content about mental health [7]. Within this landscape, mental health influencers are emerging as a topic‐based category, which includes medical professionals, therapists, behavioural coaches and laypersons who share information about mental health issues. These influencers are positioned variously within the mental health context and may act as role models or sources of telepsychology, providing information and advice and shaping opinions about mental health.

Storytelling is a well‐recognised practice within areas such as health communication, psychology, counselling and expressive therapies for identity work accomplished through interaction with others [8, 9]. However, the frameworks for describing the types of stories used by those with lived experience of mental illness and recovery, and practitioners working in educational contexts, have developed largely separately and have not yet considered the persuasive forms of storytelling used by influencers. Importantly, the ‘collapsed contexts’ [10] of social media sites mean that audiences who might engage with mental health influencers of different kinds are no longer segmented. Those seeking information, help and support about mental health may encounter stories told by a professional, a wellness figure or an influencer sharing their ‘recovery’. Furthermore, the mediated affordances of camera‐first platforms have reconfigured stories about mental health, especially through the short‐form videos that typify sites like YouTube, Instagram and TikTok. As such, we do not yet know how the stories told by the different types of mental health influencers use the multimodal contexts of social media. Nor do we know how these multimodal formats foster the information, support and solutions needed for mental health literacy.

TikTok has seen a rapid rise in use among 15–25‐year‐olds in the United States to over 75% in 2024 [11]. ‘Mental Health TikTok’ contains a wealth of content, with hashtags such as #Mentalhealth used for over 18 million posts at the time of writing. While there is emerging interest in the use of TikTok as a site for sharing information about mental health in non‐traditional ways [12], to date, there has been little narrative analysis of how this information has been shared.

In this paper, we provide the first account of how influencers presenting with lived experience of mental health challenges alongside practitioners in medical health and wellness reshape mental health narratives in the context of TikTok. This narrative approach critically evaluates the end to which storytelling about mental health is put in the fast‐changing landscape of social media.

### 1.1 Literature Review

Work in narratives related to health and mental health share common foundations, namely work by Kleinman [13], Frank [14] and Hydén [15], who helped reposition narratives as stories that articulate not only suffering but also illness as it is experienced by sufferers.

With respect to mental health, this ‘narrative turn’ [16] has informed two separate strands of research. First, the use of storytelling by practitioners and educators in the health sector has cohered into the field of narrative medicine [17, 18], whereby narratives are used as a training tool across a range of medical fields, including in psychiatry [19], family medicine [20], surgery [21] and social work [22]. Multiple systematic reviews highlight narrative medicine as improving empathy and understanding in medical practitioners [23]. Second, on lived experience narratives, most recently on the formulation of recovery by patients as a way of processing and re‐contextualising mental ill‐health [24, 25]—an emergent body of work bedevilled by low quality and modest online access history [26, 27].

Both strands of research focus on mental ill‐health and storytelling and include an occasional focus on visual elements (see, e.g., Vansteenkiste et al. who explore photovoice in recovery narratives [28] and Duong et al. who investigate comic book formats as ‘graphic medicine’ [29]). However, these developments have been largely separate and mostly focused on traditional, verbal narratives.

Recently, research has turned to narratives of mental health on online platforms. YouTube has seen multiple studies of medical narratives, including female anorexia stories [30], medical education materials [31] and male eating disorders content [32]. Instagram has been at the forefront of multimodal studies of medical conditions, including eating disorders [33, 34, 35] and depression [36]. Studies of Twitter and Facebook have been somewhat limited, but include Pendse et al.'s evaluation of ‘death language’ on Twitter [37] and Yeo's analysis of a public Hong Kong ‘School Secrets’ page on Facebook [38]. As a relatively new site, TikTok has become a popular space for examining youth mental health, as seen, for example, in studies of mental health professionals [39], child and adolescent mental health services [40] and broader analysis of #teenmentalhealth [41]. It has also featured strongly in driving mental health campaigns to support young people's mental health literacy, as in the recent www.whatsupwitheveryone.com campaign with Aardman [42, 43, 44].

Despite the multimodal affordances of social media platforms, the majority of studies of mental health in these spaces are still largely focused on ‘text‐based groups and communities’ [45]. Studies that consider data from YouTube, Instagram or TikTok tend to incorporate multimodal discussion more frequently, but many studies still opt to render the data as text‐only transcripts (see, e.g., Malova and Dunleavy's evaluation of male ED on YouTube [32] and Talbot et al.'s analysis of Orthorexia on Instagram [35]). The smaller number of studies that do contend with multimodality do so through unstructured or semi‐structured content analysis [41].

TikTok represents a unique opportunity to understand the production of mental health narratives and their role in mental health literacy. Influencers in the mental health space include two types analogous to their offline predecessors: medical professionals advancing mental health literacy in a manner typical of narrative medicine [39] and people presenting recovery narratives of lived experiences of mental illness [46]. However, a third category of influencers, described in the research literature as digital ‘gurus’ [47] and positioned in the holistic health sector, also tell stories about mental health. Earlier work has found that narratives showing ‘healthy behaviour’ often carry a persuasive effect on the audience, especially when presented with high levels of emotive language [48], a style that is highly typical of influencer marketing [49].

Previous studies of storytelling on TikTok have begun to detail the content of professionals [7, 39] and the lived experience of influencers [40], as well as more mixed types [41]. Narratives focused on wellness have received less scrutiny, although, in marketing analysis, research has explored sales effectiveness in relation to parasocial relationships [50] and open disclosure [49].

## 2 Data and Methods

### 2.1 Patient and Public Involvement

Twelve young people with lived experience of mental health challenges, aged between 16 and 25, were recruited through a campaign led by The McPin Foundation to form the young people's advisory group (YPAG) for the project. This age range incorporates adolescents and ‘emerging adults’ [51] who are likely to experience a range of life transitions and encounter challenges in mental health. The group met remotely four times during the study to help define the categories of influencers, refine the narrative categories and visual formats for the code book and discuss data examples openly to guide the analysis. Two members of the YPAG were trained and participated as coders in the inter‐rater reliability process.

### 2.2 Influencer Categories

The influencers in the data were categorised based on the information provided in their TikTok profile and linked information as:

- Health professionals who treat mental conditions, that is, psychologists, psychiatrists, therapists, counsellors and therapeutic coaches.
- Wellness figures, that is, nutritionists, fitness and well‐being coaches and alternative and complementary health practitioners.
- Lived experience experts, that is, laypersons without professional credentials or roles, documenting their journey through a range of mental health challenges, including eating disorders, obsessive‐compulsive disorder, anxiety and depressive disorders, bipolar disorders, borderline personality disorder, complex post‐traumatic stress disorders and dissociative disorders.

The categories were derived and refined from the YPAG's recommendations for content that they considered relevant to young people. The profile information was used to categorise individual accounts (e.g., via self‐presented professions, credentials or positioning in usernames with terms such as ‘recovery’ or ‘healing’). Where possible, the status of health professionals was further checked via their linked information. 90% (*n* = 27) of categorised health professionals gave confirmation of license to practice. There was no overlap in the influencer categories. One borderline case (therapeutic or wellness coaching) was discussed and resolved based on the language used in the influencer's profile and the type of content posted.

Influencers were further classed according to follower size as micro (10,000+ followers), macro (100,000+ followers) or mega (1,000,000+) [52] and care was taken to ensure a balanced range of follower sizes across categories. Nano influencers (< 10,000 followers) were excluded.

### 2.3 Data Extraction and Selection

Data collection followed a seed‐based model, with the initial influencer accounts recommended by the YPAG. Next, we extracted video and profile descriptions and used the highest‐frequency keywords to broaden our profile selection using TikTok's Research Application Programming Interface (API). We limited our profile selection to English‐speaking countries (the United States, the United Kingdom and Australia) and a minimum of 10,000 followers. In accordance with TikTok's Terms of Service, all illustrations in this paper are composite line drawings rather than reproductions of content from individual creators.

The data extracted from TikTok included all publicly available videos from February 2022 to March 2023, totalling over 27,000 videos about mental health from 90 users across the three influencer categories. Video transcriptions were collected verbatim through the ‘voice to text’ function of the TikTok API, which includes auto‐generated, user‐edited and user‐generated subtitles as found in the videos.

A smaller sample for qualitative analysis was selected using keyword criteria. Any video included the terms ‘mental health’ and ‘therapy’ or used the hashtag ‘#mentalhealth’. We further selected videos that had gained at least 20 likes. No more than 10 videos per account were included. This yielded 660 videos from 66 users, from which a minimum of five videos per user were analysed. The final data sample consisted of 154 videos from mental health professionals, 126 videos from lived experience influencers and 118 videos from wellness practitioners. Python was used to process and reformat the data to allow analysis of the video transcriptions in Nvivo 14 [53].

### 2.4 Analysis

The mixed‐methods discourse analysis used a small‐stories approach [54] that considered the verbal and visual choices as formatted ‘ways of telling’. In line with mediated narrative analysis [55], we retain eventhood as a core characteristic of narrative, distinguishing between events that are represented as taking place in the past, present, habitually or as decontextualised cause–effect patterns without a temporal context. The categories for visual formats were based on the recognisable practices observed in the data. The labels for each category were agreed upon in discussion with the YPAG.

The coding guidelines, produced by Author 6, emerged inductively through partial, continuous analysis of the data. Narrative types (Table 1) were coded at a sentence level in Nvivo, while visual formatting (Table 2) was coded on a case‐level basis with each video using one or more visual formats. The full codebook, including textual and visual examples, has been made publicly available through the Open Science Framework [56].

**Table 1:** Simplified description of the narrative types from the codebook.

Narrative type	
Present tense stories	The events in the story are presented as if taking place the day the content was posted, indicated by temporal adverbs like ‘just’, ‘right now’
Past tense stories	The events in the story took place in the past, indicated by past tense verbs and temporal adverbs like ‘last night’, ‘a week ago’
Habitual scenarios	The events are presented as typical or regularly occurring, indicated by temporal adverbs like ‘every time’, ‘whenever’
Explainers	Mental health symptoms, causes and solutions are narrated as cause and effect, often through ‘if’ or ‘when…’ formulations that depict typical event sequences
Intertextual stories	Quotes or sound clips from popular media are used to convey past mental health experiences

**Table 2:** Simplified description of the TikTok visual formats from the codebook.

Visual format	
Duet	The creator's video appears side‐by‐side with a video from another creator playing simultaneously
Stitch	The creator adds a response to a pre‐existing TikTok video
Greenscreen	A background image or video is used as a backdrop for the post
Enactment	The creator performs a ‘skit’ or dialogue in multiple shots
Watch as I do this	The creator is shown actively engaged in an activity (e.g., cooking, exercising and applying make‐up) while talking to the camera
Talking head	The creator appears in the video talking directly to the camera with no additional activity
Compilations	The video consists of multiple images or clips assumed to unfold over time, considering, e.g., changes in setting or creator's appearance

To ensure the reliability of the codes, two subject experts within the research team and two members of the YPAG performed inter‐annotator testing of a randomised 10% sample of the data. On the first round of coding, the levels of agreement for each of the codes were as follows: narrative types (95% agreement, Krippendorff's *α* = 0.729) and visual formats (80% agreement, *α* = 0.592). After review, the disagreements related to lack of clarity for the Compilations and Watch as I do this. The criteria for these categories were revised, and the second round of coding improved agreement for the visual categories to 98% (*α* = 0.956). Coding of the full sample in Nvivo was then completed by Author 6.

The outputs from NVivo were analysed to identify the combinations of narrative type and visual format adopted by the three categories of influencers in our dataset. Further qualitative analysis of the verbal‐visual ‘ways of telling’ was undertaken by the two subject experts from the research team. The discourse‐analytic interpretation of the storytelling styles focused on the ways in which the affordances of TikTok as a site for telling were used to remediate earlier narrative genres of mental health and the identities of the tellers that emerged therefrom.

## 3 Quantitative Results

**Figure 1:**
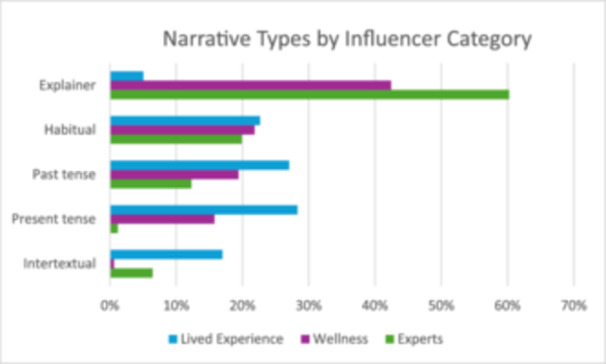
Bar chart showing the frequency of narrative types by influencer category.

Overall, the 398 videos contained 495 narratives. Explainers were most frequent (37%, *n* = 181), followed by habitual scenarios (21%, *n* = 106), past tense narratives (19%, *n* = 96), present tense (15%, *n* = 73) and intertextual stories were least frequent (8%, *n* = 39). The narrative type varied across the influencer categories. See Table 3 and Figure 1.

**Table 3:** Frequency of narrative types according to influencer categories.

Narrative type	Health professionals		Lived experience		Wellness	
Present tense	2	1%	45	28%	26	16%
Past tense	21	12%	43	27%	32	19%
Intertextual	11	6%	27	17%	1	1%
Habitual scenario	34	20%	36	23%	36	22%
Explainer	103	60%	8	5%	70	42%
Total	171	100%	159	100%	165	100%

Explainers were used most often by health professionals and least by lived experience influencers. Conversely, lived experience influencers used the present tense, past tense and intertextual stories more often than did the health professionals. Wellness influencers occupied a mid‐position, using explainers the most often and habitual, past and present tense stories less often. The results suggest a contrast in how the different types of influencers narrated mental health information and experiences. Lived experience influencers used personal experiences of mental health and illness, while health professionals and wellness figures ‘explained’ mental health symptoms, conditions and treatments, drawing on personal narratives to a lesser extent.

The visual formats used in the videos also varied. The default format showed the influencer as a static ‘talking head’ in over two‐thirds of the videos (67%, *n* = 252). In the remaining videos, the creators used the editing formats of TikTok, most often by compiling clips in a series (13%, *n* = 47). Greenscreen formats were used in 6% (*n* = 24) of the videos. A similar percentage of the videos used a ‘watch as I do this’ format, where the content creator performed a task (*n* = 21). Influencers also enacted ‘skits’ in a small number of videos (3%, *n* = 12). A similar percentage accounted for the use of stitches (3%, *n* = 13), while the least frequent format was the duet (2%, *n* = 6).

**Figure 2:**
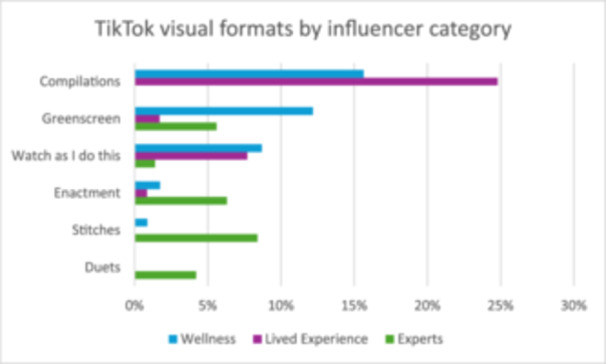
Frequency of visual formats by influencer category (excluding ‘talking head’).

The extent to which the categories of influencers used the default static, ‘talking head’ format was largely similar. Health professionals used this most often (74%, *n* = 106), while lived experience and wellness influencers used this less (65%, *n* = 117% and 61%, *n* = 115). As seen in Figure 2, the frequency of the edited formats varied by influencer category. Compilations were used most often by lived experience influencers (25%, *n* = 29), to a lesser extent by wellness influencers (16%, *n* = 18) and not at all by health professionals. Conversely, stitches and duets were used more often by the experts (9% combined *n* = 18), seldom by the wellness influencers (once) and were never used by the lived experience influencers. Greenscreen and the ‘Watch as I do this’ format were used most often by wellness influencers, in 12% and 9% of their videos, respectively. Lived experience used ‘Watch as I do this’ formats in a similar amount to the wellness figure (8%, *n* = 9), while this occurred only once in the health professionals' data. Instead, health professionals also used Greenscreens in 6% (*n* = 8) of their data, while this occurred only twice in the lived experience videos.

## 4 Qualitative Discussion

The combination of visual and verbal formats gives rise to three distinct storytelling styles, used variously by the influencers in each category.

### 4.1 Lived Experience Influencers

The verbal‐visual storytelling combinations used by lived experience influencers remediated various types of recovery narratives documented in earlier literature [25, 26].

**Figure 3:**
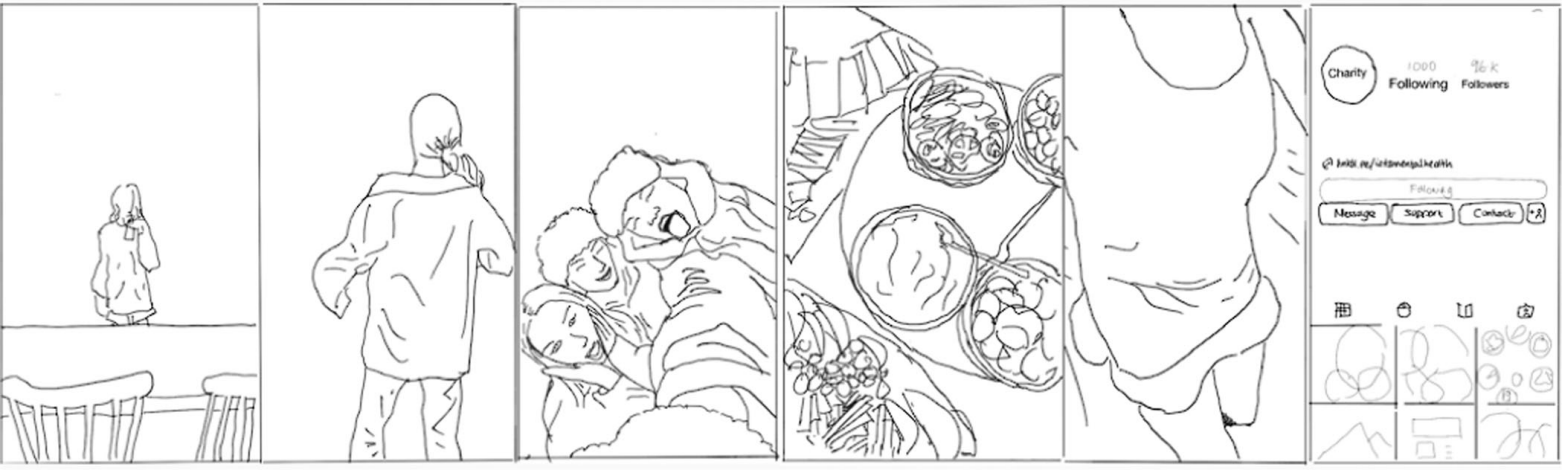
Line drawing illustrating a compilation used with a past tense narrative.

First, stories of ‘enlightenment’ [26] that showed the influencers' progression towards health were told in past tense narratives combined with compilations (see Figure 3, showing the influencer's changing experience of food, health and social engagement). Other types of recovery, such as ‘endurance’ and ‘endeavour’, were told in present tense narratives using compilations to depict the influencer's fluctuating experiences in stories of ‘making progress’ or ‘surviving day to day’. These compilations drew on vlog formats of the ‘Day in the Life’ and the ‘Food Journal’ [57] in trends such as ‘Day in the Life on a Psych Ward’ or ‘What I eat in a Day’.

**Figure 4:**
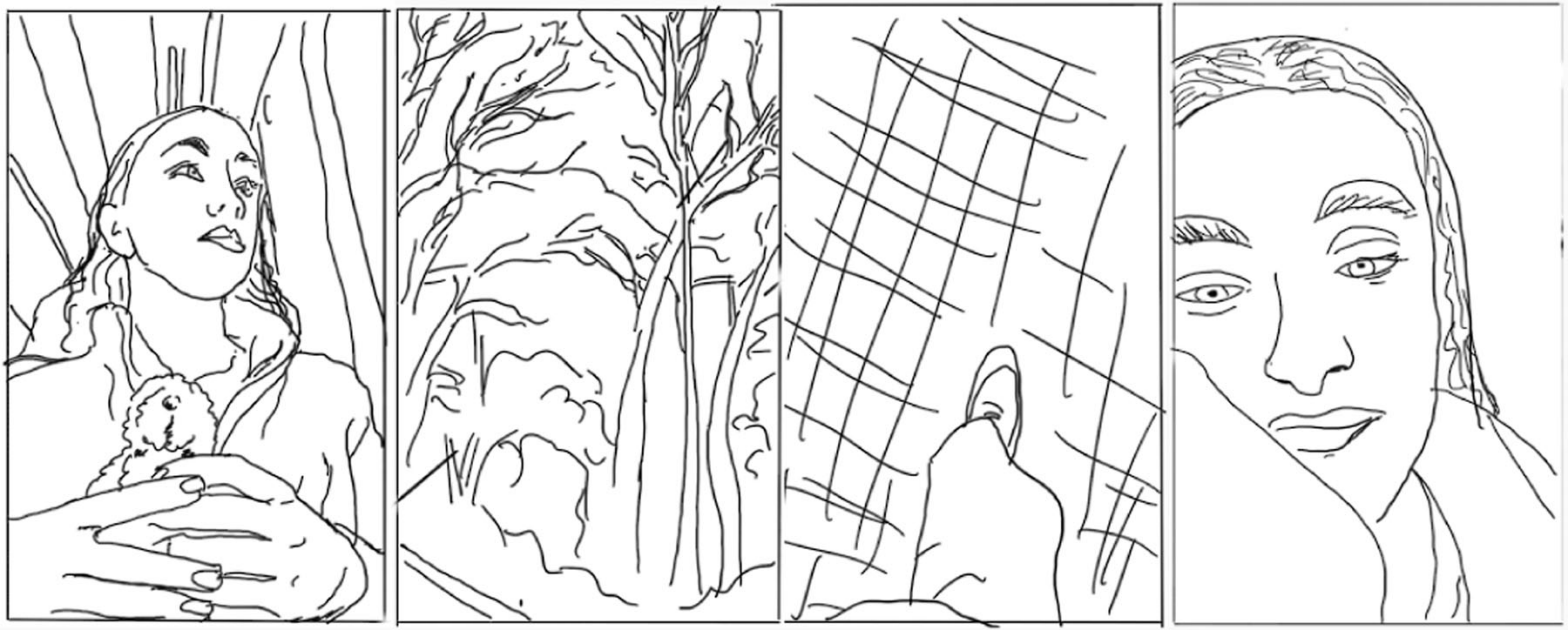
Line drawing illustrating a compilation using visual positioning to ‘look with’ the influencer (in Frames 2 and 3) and close‐ups.

In all three types of recovery narrative, the intersubjective positioning used in the compilations encouraged the audience to move between ‘looking at’ and ‘looking with’ the influencer [58], constructing a shared experience oriented to the influencer's standpoint. The visual content also included close‐up shots of the influencer's spontaneous, tearful reactions or shifts in mood, depicting the ups and downs of living with mental health conditions. See, for example, Frames 2 and 3 in Figure 3 and Frame 4 in Figure 4.

**Figure 5:**
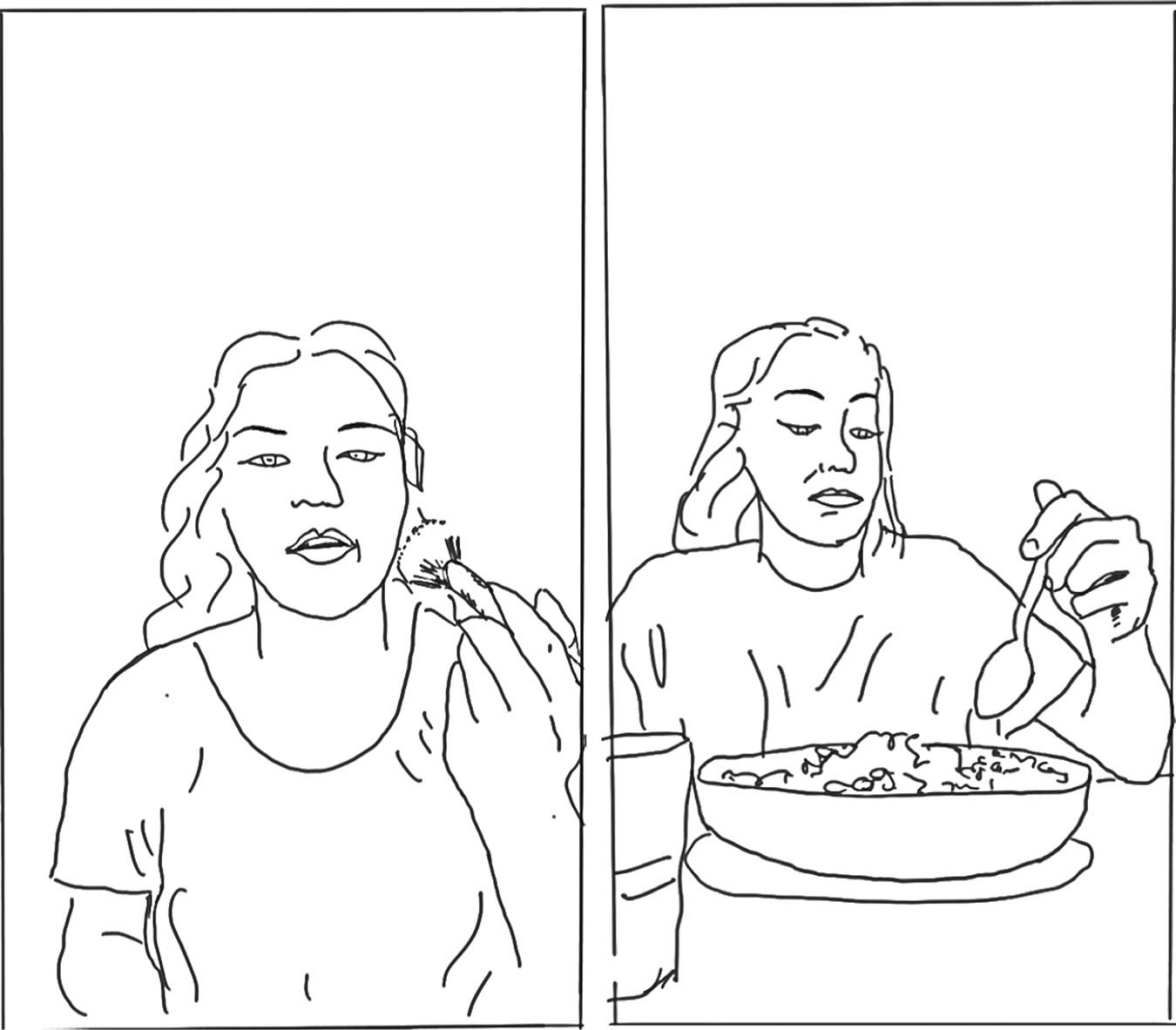
Line drawings showing ‘Watch as I do this’ formats used by lived experience influencers. On the left, a ‘Get ready with me format’ and on the right a ‘What I eat in a day’ format.

Lived experience influencers also told recovery narratives in ‘Watch as I do this’ formats, such as the ‘Get ready with me’ and ‘What I eat in a day’ formats shown in Frames 1 and 2 of Figure 5. These formats are similar to the ‘chatty vlog’ [57], showing the influencer using private or semi‐private spaces such as a bedroom, bathroom or kitchen as an everyday context for telling stories of endeavour and endurance about their mental health. The visual aspects of narration performed authenticity, granting the audience apparent backstage access to the influencer's life.

Not all recovery stories move towards resolution. So‐called ‘untellable stories’ [25] of struggle, crisis and relapse were told by lived experience influencers in their intertextual stories. These videos showed the influencer lip‐syncing to sound clips that used in‐group references to popular culture to infer challenging moments in the mental health experience (e.g., when therapeutic treatments have failed). The emotional tonality conveyed through the semiotic resources varied, suggesting melancholy, dark humour and irony to frame the difficult aspects of mental health that formed the focus of these stories.

The multimodal affordances of TikTok's short‐form videos were used by the lived experience influencers to develop earlier forms of recovery narratives in three key ways. First, they used visual intersubjectivity to project shared perspectives on mental health experiences. Second, they used everyday settings and recognisable social media genres to normalise a range of positions towards recovery. Third, they conveyed ‘untellable’ stories through intertextual sound clips drawn from in‐group popular culture references. Unlike earlier thematic analyses of verbal recovery narratives, the multimodal analysis brings to light the ways in which short‐form videos increase shared experience and peer support between tellers and their audience.

### 4.2 Mental Health Professionals

The verbal‐visual styles of the influencers with professional mental health credentials remediated the use of storytelling as a form of scientific explanation [59] and the educational practices of narrative medicine [18].

**Figure 6:**
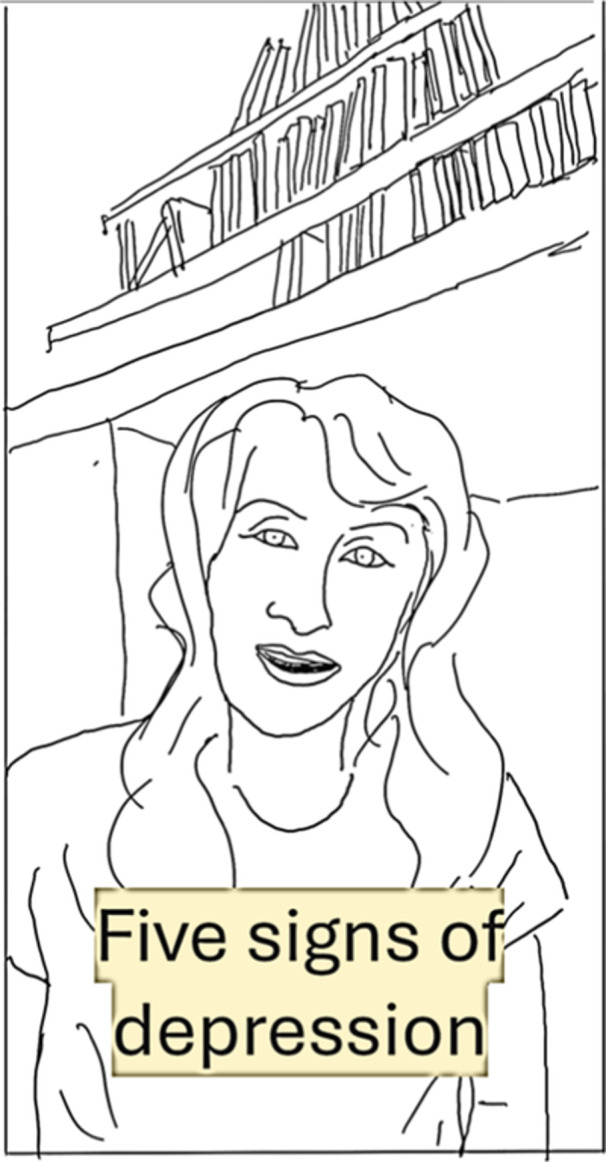
Line drawing showing a ‘talking head’ format with a listicle explainer caption.

Mental health professionals used explainers (cause‐and‐effect narrative patterns) to inform their audience about the symptoms of mental health conditions and treatments. Typically they used a ‘talking head’ format, as seen in Figure 6, where the influencer gazed directly into the camera to address a general audience in a form of visual synthetic personalisation [60]. The multimodal characteristics of these videos projected expertise via settings or props indicative of a professional context (e.g., with bookcases in the background) or a therapist's lounge. Visual captions were added to some videos to convey the creator's professional status, such as ‘licensed therapist’. Other captions used journalistic genres such as the listicle to narrativise selected symptoms and experiences of mental health, visually counting ‘signs’ of mental health issues, symptoms or facts about a disorder or treatment.

**Figure 7:**
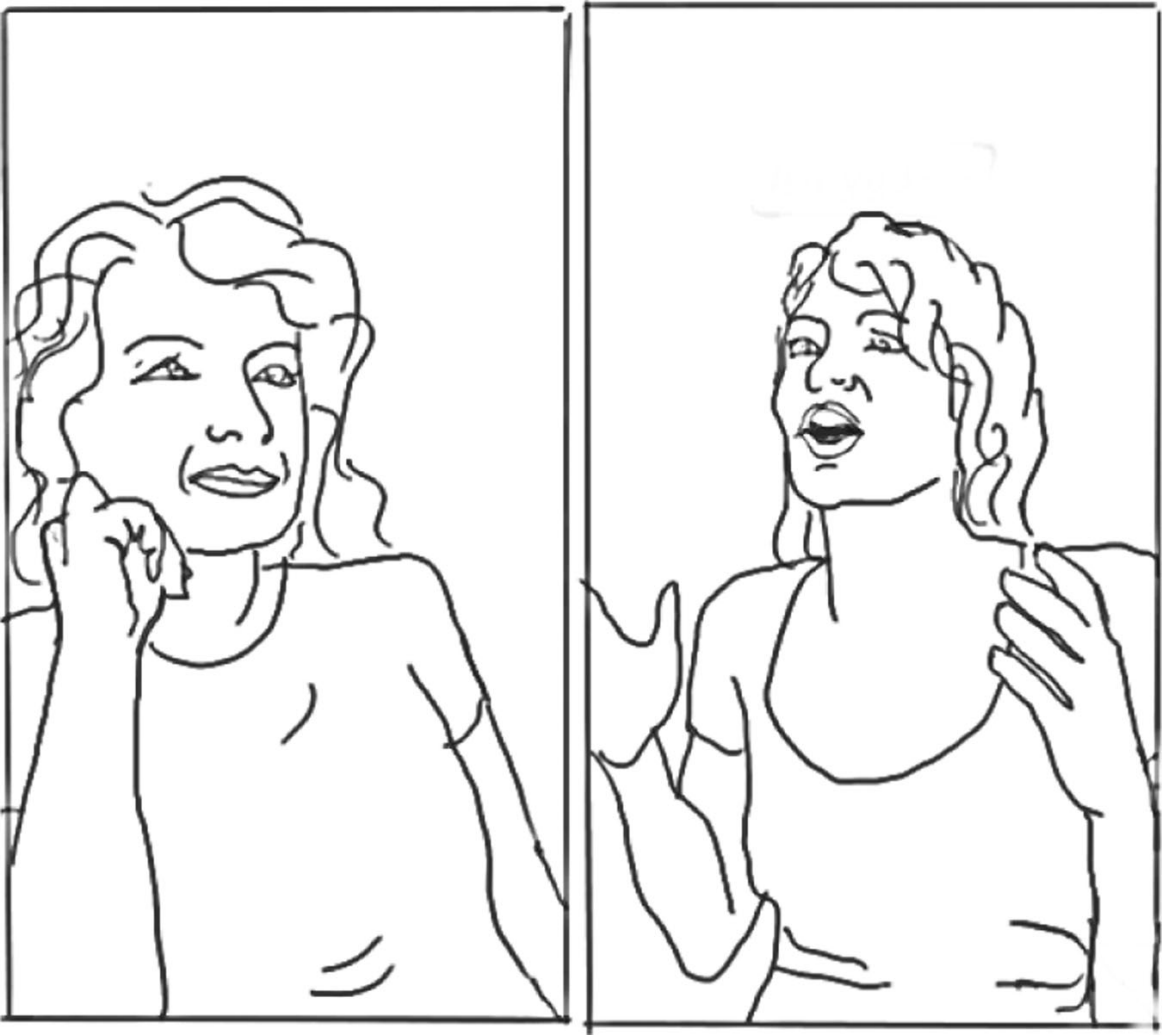
Line drawing showing a therapist enacting a conflict with a client.

The narrativisation of mental health conditions was also remediated by the professional healthcare influencers who used enactments to inform their audiences about the processes in talking therapy, dramatising the assumed psychological processes experienced by the patient, or the interactions between a patient and practitioner. The contrasting perspectives of the patient and therapist are conveyed visually through the alternating postures and paralinguistic features performed by the content‐creator in the sequence of headshots, often using further captions and sound clips to animate the emotional and cognitive characteristics of psychological challenges (see Figure 7).

Earlier studies of narrative medicine documented the use of close reading in a ‘read‐reflect‐respond’ model [23]. TikTok's response videos (stitches and duets) provided health professionals with a new, multimodal format for similar responsive commentary to stories produced by other content creators. Unlike the empathetic motivations of narrative medicine and its non‐hierarchical impetus [18], the professionals took varying, often critical, stances towards the original content produced by laypersons in TikTok and capitalised on the visibility afforded to both the original and response video in stitches and duets to further promote their own position as authoritative sources of mental health information. In some cases, the stitches were used as telepsychology, offering advice to the content creator, typically coupled with a marketing call to action to engage the influencer's services.

In sum, the multimodal affordances of the short‐form videos used by the health professionals performed their expertise through visual cues and captions, whilst shifting their communicative style to the lay knowledge invoked by listicles and enactments. In contrast to narrative medicine, where stories are used in pedagogic practice to support other professionals, the critical commentary offered by these influencers, along with marketing calls for action, suggests the commercialisation of these stories to attract new audiences and future clients.

### 4.3 Wellness Influencers

The multimodal formats used by the wellness influencers drew on storytelling styles found in advertorials [61], blending informative and commercial genres to convey information about mental health and persuade their target audiences of the benefits found in the goods and services that they promoted as influencers.

Narratively, wellness influencers drew primarily on explainers (*n* = 70), which, combined with habitual narratives, created cause‐and‐effect sequences used to explain mental health issues. Verbally, this is similar to the narrative patterns of health professionals. Visually, however, wellness influencers drew on a more varied set of formats, mirroring variously the lived experience use of compilations (*n* = 18) and ‘Watch as I do this’ (*n* = 10) to create a ‘shared experience’, and the health professional use of greenscreen overlays to project expertise (*n* = 14).

**Figure 8:**
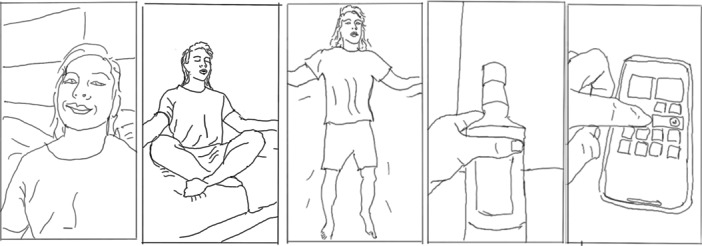
Line drawing showing a compilation from a wellness influencer demonstrating healthy activities, inviting a shared perspective in Frames 4 and 5.

The compilations showed sequenced images of the influencer performing the generic well‐being activities of an ‘ideal day’, exercising, meditating, making healthy food choices, reducing screen time or regulating sleep (see Figure 8).

**Figure 9:**
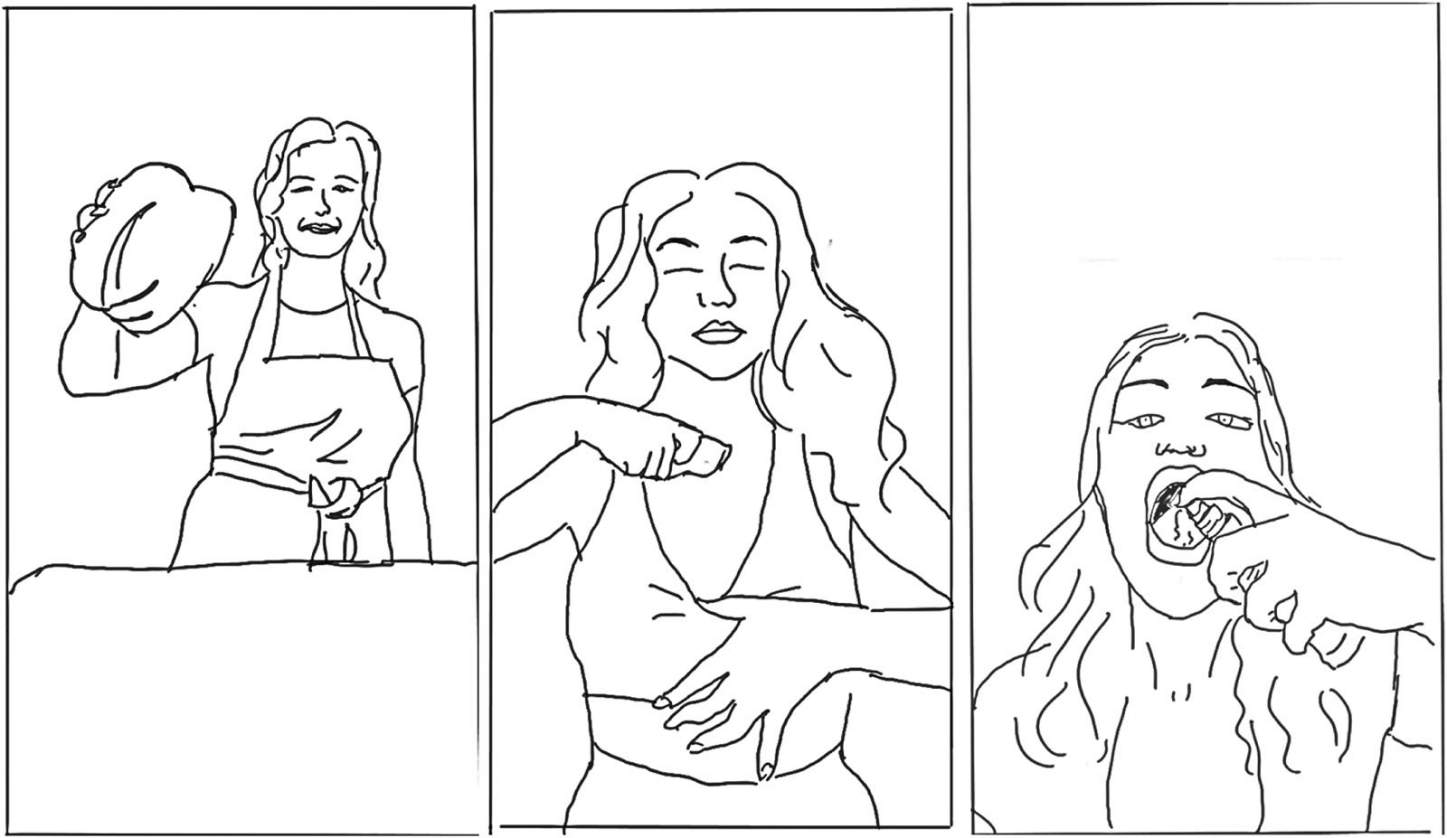
Line drawings showing three wellness influencers using the ‘Watch as I do this’ format, from left to right, preparing healthy food, using a gua sha massage technique and taking supplements, respectively.

The visual genres used by the wellness influencers in their ‘Watch as I do this’ videos performed ‘ordinary expertise’ [62] through tutorial formats that demonstrated how to undertake the solutions for mental health issues. For example, the influencer showed how to prepare a healthy meal, employ a holistic technique, exercise or take supplements (see Figure 9). Verbally, these videos used explainers, including listicles which diagnosed generalised mental health issues (anxiety and stress) but proposed non‐medical solutions to treat them, for example, via diet, exercise, supplements or holistic therapy, often adopting pseudo‐scientific rhetoric. Like health professionals, these wellness influencers ended their stories with a marketing call to action that invited further engagement, for example, to ‘Send a DM’ to engage with content or services from the influencer.

The commercial implications of the advertorial stories told by wellness influencers are seen in their use of the Greenscreen format. Greenscreen techniques were used by the influencers to display a visual background overlaid with a cut of their ‘talking head’. The backdrops in videos included screenshots of the influencer's lay summaries of scientific reports, images of the influencer using a particular treatment or demonstrating outcomes with transformation videos. Most often, the screenshots included images of the online stores where products could be purchased, coupled with recommendations: ‘You can find this product at [store] and also find them on Amazon.’

The multimodal formats used by the wellness influencers positioned them as role models who demonstrated activities and recommended resources as solutions leading to positive mental health and well‐being. This was coupled with promotional discourse, showing purchase options or inviting engagement with the influencers' own services, demonstrating the commercialisation of mental health information and support by this means.

## 5 Conclusion

The three categories of influencers (health professionals, wellness and lived experience) used multimodal storytelling styles that variously remediated earlier practices used in narrative medicine, marketing and peer support via recovery narratives. The analysis shows how differences are amplified between the storytelling practices of health professionals, who educate others through talking head explainers, enactments and stitches, and lived experience influencers, who invited shared perspectives on their stories of illness, treatment and recovery through compilations and ‘Watch as I do this’ formats. The multimodal aspects of our analysis also shed light on important points of convergence between the wellness practitioners who occupy an interdiscursive mid‐space, blending the verbal aspects of ‘informing’ (explainers) akin to the health professionals with the visual narration of lived experience (compilations and watch as I do this), to promote solutions through recommendation and advertising. The calls to action in the data suggest the commercialisation of mental health content from both medical and alternative health figures in social media.

### 5.1 Strengths and Limitations

The size of our dataset and unique research design, comparing three categories of influencers, is a strength. The involvement of the YPAG in our research design and analysis of the data increased the credibility of our results and the relevance of the findings for the mental health needs of young people in the United Kingdom. There were some limitations. The data was limited to English Language regions only, but since we did not further subdivide the influencers by nationality, such intercultural differences remain to be explored. The analysis focuses only on the videos, not on response or reception.

### 5.2 Implications

The implications of our results are twofold.

First, the contrasting storytelling styles used by lived experience and health professional influencers perpetuate a gap between the aspects of mental health literacy as communicated by social media influencers. The ‘informing’ style of the professionals may be useful for conveying generalised knowledge about mental health and illness. Conversely, the ‘sharing’ style used by lived experience influencers fosters peer support, with little information about conditions or treatments offered. The gap between the modes through which information and support are provided may mean that the mental health literacy developed via these sources is partial and imbalanced.

Second, the multimodal overlaps between the verbal components of the ‘informing’ and the visual components from the ‘sharing’ styles of storytelling co‐opted by the wellness influencers suggest visual literacy is an important aspect of evaluating mental health resources. Content that appears similar may mimic authoritative and authentic communication but promote non‐medical solutions for mental health that are unsupported by evidence.

Further research should explore how young people engage with content shared by influencers about mental health, acknowledging the benefits and further challenges this type of health communication has for their mental health needs. There is a need for further education so that young people can navigate this unregulated source of information more effectively.

## Author Contributions

**Alex Christiansen:** conceptualisation, methodology, software, data curation, formal analysis, writing – review and editing, writing – original draft. **Shioma‐Lei Craythorne:** formal analysis, conceptualisation. **Paul Crawford:** conceptualisation, supervision, funding acquisition, writing – review and editing. **Michael Larkin:** conceptualisation, funding acquisition, supervision. **Aalok Gohil:** formal analysis. **Spencer Strutt:** formal analysis. **Ruth Page:** conceptualisation, methodology, supervision, formal analysis, validation, investigation, funding acquisition, visualisation, project administration, resources, writing – review and editing, writing – original draft.

## Ethics Statement

The research was approved by the Humanities and Social Sciences (HASS) ethics committee at the University of Birmingham and the University Research Integrity and Ethics Committee (URIEC) at Aston University.

## Conflicts of Interest

The authors declare no conflicts of interest.

## Data Availability

The data used in the research were made available through the TikTok Research API, and restrictions apply to the direct availability of the data on the basis of the Terms of Service provided by TikTok. Precise instructions for regenerating the data used in the study are available upon request from the corresponding author (Alex Christiansen).
